# Evaluation of suitable reference genes in *Brassica juncea* and its wild relative *Camelina sativa* for qRT-PCR analysis under various stress conditions

**DOI:** 10.1371/journal.pone.0222530

**Published:** 2019-09-20

**Authors:** Shikha Dixit, Vinod Kumar Jangid, Anita Grover

**Affiliations:** Plant-Pathogen Interaction Laboratory, National Institute for Plant Biotechnology, Pusa Campus, New Delhi, India; Universidad Politecnica de Cartagena, SPAIN

## Abstract

Quantitative real-time PCR (qRT-PCR) is an efficient method to estimate the gene expression levels but the accuracy of its result largely depends on the stability of the reference gene. Many studies have reported considerable variation in the expression of reference genes (RGs) in different tissue and conditions. Therefore, screening for appropriate RGs with stable expression is crucial for functional analysis of the target gene. Two closely related crucifers *Brassica juncea* (cultivated) and *Camelina sativa* (wild) respond differently towards various abiotic and biotic stress where *C*. *sativa* exhibits higher tolerance to various stress. Comparative gene expression analysis of the target genes between two such species is the key approach to understand the mechanism of a plant’s response to stress. However, using an unsuitable RG can lead to misinterpretation of expression levels of the target gene in such studies. In this investigation, the stability of seven candidate RGs including traditional housekeeping genes (HKGs) and novel candidate RGs were identified across diverse sample sets of *B*. *juncea* and *C*. *sativa* representing- hormone treated, wounded, *Alternaria brassicae* inoculated and combination treated samples (exogenous hormone treatment followed by *A*. *brassicae* inoculation). In this investigation, we identified stable RGs in both the species and the most suitable RGs to perform an unbiased comparative gene expression analysis between *B*. *juncea* and *C*. *sativa*. Results revealed that *TIPS41* and *PP2A* were identified as the overall best performing RGs in both the species. However, the most suitable RG for each sample subset representing different condition must be individually selected. In Hormone treated and wounded samples *TIPS41* expressed stably in both the species and in *A*. *brassicae* inoculated and combination treatment performance of *PP2A* was the best. In this study, for the first time, we have identified and validated stable reference gene in *C*. *sativa* for accurate normalization of gene expression data.

## Introduction

Quantification of mRNA transcript levels provides important insights into the intricate metabolic pathways and signaling networks underlying the plant’s physiological plant’s physiological response to various abiotic and biotic stresses. qRT-PCR is a sensitive, accurate and cost-effective method to evaluate the expression of the target gene in different tissues, organs and treatments [1,2]. However, the accuracy of the expression data is affected by many factors, such as RNA quality, purity, PCR amplification efficiency, technical and true biological variations [3,4]. To control these variables and avoid bias in qRT-PCR, selecting stably expressed RGs under different experimental conditions are crucial for normalization. Selecting an inappropriate RG leads to misinterpretation of the expression levels of the target gene [5,6]. In theory, RGs are the genes which maintain a constant expression level in all cell types and under every experimental condition. However, many previous studies have reported that based on the experimental conditions and species, the expression level of the traditionally used RG is variable [7–9]. The housekeeping genes are required to maintain basic cellular functions such as components of the cytoskeleton, vesicular transport, cell cycle, glycolytic pathway, protein synthesis, and protein degradation. Traditionally, actin (*ACT*), elongation factor 1- alpha (*EF1A*), polyubiquitin (*UBQ*) and tubulin alpha (*TUA*) are used as RGs in expression analysis [10–12]. In recent years with the development of microarray and Affymatrix GeneChip many novel genes have been identified as suitable RGs and were found to exhibit stable expression in many species like *Arabidopsis thaliana* [13], *B*. *juncea* [14] *Brassica napus* [15], tobacco [16] and Rice [11] Moreover, there is evidence to suggest that the expression of HKGs is differentially regulated under variable experimental conditions and species [17–19]. Therefore, selecting stably expressed RG is crucial for accurate normalization of gene expression data.

In the past years, many statistical algorithms like geNorm [20], Normfinder [21] and BestKeeper [22] had been developed to simplify the selection of appropriate RGs. geNorm gives expression stability value (M) of each RG under a given experimental condition. M value is the average pairwise variation in the expression of particular RG with all the other RG analyzed in the experiment. Gene exhibiting lower M value have more stable expression and higher M value depicts less stable expression of RG in a given set of samples. geNorm also determines the minimum number of RGs required for accurate normalization by calculating normalization factors (NFs) with a cutoff value for Vn/n+1 as 0.15, below this value no additional RG is required for normalization. NormFinder evaluates expression stability of RGs with a different mathematical model wherein inter- and intra- group variation in the expression of candidate RGs is taken into consideration. This algorithm assigns an expression stability value to the RGs. A lower value is the determinant of higher expression stability and lower inter and intra group variation. In the BestKeeper tool, the original Ct values are used for identification of the most stable RG under a given experimental condition.

The *Brassicaceae family* comprises of many prominent species with great agronomical importance, Indian mustard (*B*. *juncea*) and false flax (*C*. *sativa*) are two such species with huge economic and agricultural value across the world. *B*. *juncea* contributes the third largest share to the global vegetable oil production after soybean and groundnut [23,24]. *C*. *sativa*, which is a wild crucifer, has recently gained increasing interest because of its favourable attributes such as resistance towards salinity [25] and drought [26] and resistance to diseases, such as blackspot [24] and blackleg [24]. The cultivated species *B*. *juncea* is a potent target of necrotrophic pathogen *A*. *brassicae* whereas, the wild type species *C*. *sativa* express resistance against the *A*. *brassicae*. It is a known fact that phytohormones like salicylic acid (SA), jasmonic acid (JA) and abscisic acid (ABA) and their cross-talk play a central role in determining the plant’s response to a particular pathogen [27,28]. To understand the mechanism of pathogen response operative in resistant/susceptible species, differential expression of target genes observed in *B*. *juncea* and *C*. *sativa* in response to various challenges is an adequate approach. Therefore, it is important to identify suitable RGs which express stably in *B*. *juncea* and *C*. *sativa* in order to perform an accurate comparative gene expression analysis in these two species. In *B*. *juncea*, efforts have been made previously to identify suitable RGs in different developmental stages and treatments [14]. However, no such normalization study has been performed so far for *A*. *brassicae* inoculated samples of *B*. *juncea*. Also, to the best of our knowledge, this study is the first attempt to identify stable reference genes in *C*. *sativa*. In the current study, we compared the performance of 7 candidate RGs across 8 sample subsets of *B*. *juncea* and *C*. *sativa* subjected to various treatments. Statistical algorithm geNorm, NormFinder and BestKeeper were utilized for the analysis. The purpose of this study was to identify stable RGs in both the species under a given experimental condition as well as to recognize those RGs which can be used for efficient comparison of target gene expression among *B*. *juncea* and *C*. *sativa*.

## Materials and methods

### Plant material and stress treatment

Mustard (*B*. *juncea*) and a wild relative False flax (*C*. *sativa*) were selected for the study. Plants were grown in a controlled environment growth chamber programmed for 16h/8h of light/dark cycle at a temperature of 24°C/20°C for day/night and relative humidity of 80%. 35 days old plants were subjected to various treatments and mature leaf tissue were collected at different time points -0, 3, 6, 12, 24, 48, 72, 96 hours after treatment (hat). Samples were snap frozen in liquid nitrogen and stored at -80°C. *B*. *juncea* and *C*. *sativa* Plants were subjected to following treatments: SA, MeJA, ABA, wounding, *A*. *brassicae* inoculation (I), SA+ I, MeJA+I and ABA+I. For hormone treatment, plants were sprayed with SA (1mM), methyl jasmonate (MeJA) (100μM) and ABA (100μM) solution. For wounding treatment, leaf surface was scratched lightly with the help of blunt forceps without breaking the leaf. For *A*. *brassicae* treatment, fungus was cultured in half strength potato dextrose agar (PDA) and spore suspension (5×10^6^ spores/ml) prepared with 20 days old culture plate was used to inoculate the plants. For combination treatment, plants were sprayed with SA, MeJA or ABA and each hormone-treated plant was inoculated with *A*. *brassicae* as described previously. For each treatment per time point two biological replicates were maintained. In total for each species 128 samples were collected representing 8 treatments at 8 different time points. Control plants were maintained simultaneously and control samples were also collected at the same time points.

### Total RNA isolation and cDNA synthesis

The TRIzol^™^ reagent (Invitrogen) was used for total RNA extraction from frozen leaf tissues. Purity and quantity of RNA were determined using NanoDrop^™^ 2000 spectrophotometer (Thermo scientific). Samples with A_260_/A_280_ > 1.8 and A_260_/A_230_ > 2 were used further for cDNA synthesis. RNA integrity of all the samples was confirmed by 2% agarose gel electrophoresis. First strand cDNA synthesis kit (Thermo scientific) was used to synthesize the first strand cDNA from 1μg of RNA for 20 μl of reaction with oligo dT primers. cDNA was diluted to 1:10 (cDNA: nuclease free water) for further qPCR reactions.

### Candidate reference genes selection and primer designing

7 candidate reference genes were selected for the experiment. Traditionally used RGs *ACT7* (actin 7), *EF1A* (elongation factor 1 alpha), *UBQ9* (ubiquitin 9) and *TUA* (tubulin alpha) were used in the study. In addition, *TIPS41* (tonoplastic intrinsic proteins 41), *CAC* (Clathrin adopter complex) and *PP2A* (protein phosphatase 2A) which showed stable expression in the previous studies in *A*. *thaliana* and other *brassica* species [13][7][14] were also included in the study. To design the primers of RGs and *PDF1*.*2* gene, *Arabidopsis* CDS sequence was obtained from Genbank and used as a query sequence in BlASTn to obtain the homologous sequences from *B*. *juncea* and its wild relatives from B. *rapa* genome portal (http://brassicadb.org/brad). To ensure positive amplification in *B*. *juncea* and *C*. *sativa*, sequences of each gene were aligned using clustalw tool and primers were designed from the consensus region of the aligned sequences. Accession numbers of *B*. *juncea* and *C*. *sativa* genes used for the clustalw alignment is provided in supplementary file S1 Doc. For all the genes, primers were designed using online tool Primer 3 [29] with following parameters: primer length 20–25 bp, amplicon size 100-200bp, GC content 60–65%, melting temperature 60–65°C, absence of hairpin structure, homodimer and heterodimer. To check the specificity of the designed primer agarose gel electrophoresis and melt curve analysis was done. Amplified products were run at 2% agarose gel and melt curve was generated following qRT-PCR in both the genotypes. Amplification efficiency (E) and correlation coefficient (R^2^) were calculated based on the slope of standard curve prepared from a series of dilutions as per following equation: E (%) = (10^−1/slope^ -1) ×100. Primer details and amplification efficiency is shown in Table 1.

**Table 1:** Description of the seven candidate reference genes adopted in the study.

Gene symbol	Gene name	Accession number	Primers sequences 5'-3'	Amplicon length (bp)	T_m_(° C) F/R	*B*. *juncea*		*C*. *sativa*	
						E* (%)	R^2^	E* (%)	R^2^
*ACT7*	Actin 7	NM_121018.4	F-GGAATCGCTGACCGTATGAG	109	60.3/60.0	99.78	0.9802	98.47	0.91
			R-ACCCTCCAATCCAGACACTG						
*CAC*	Clathrin adaptor complex	NM_124033.4	F-TTGAAGTTGGGGTTGAATGA	150	58.9/58.9	100.69	0.9926	99.48	0.9207
			R-AACAGTCTTCTCGGAGTTGAATC						
*EF1A*	Elongation factor 1 alpha	XM_010477272.2	F-CCCTCCGTCTACCACTTCAG	101	59.7/59.6	96.33	0.9994	99.33	0.9475
			R-CACAACCATACCAGGCTTGA						
*PP2A*	Protein phosphatase 2A	BT000108	F-GTCAACAATCCGCACTACCTACA	111	61.3/61.8	100.2	0.9759	103.83	0.9515
			R-CAACCACGACGGGAAGAAAC						
*TIPS41*	Tonoplastic intrinsic protein 41	NM_119592.5	F-GGTTGAGAGAGACGAGAATGC	118	59.7/59.5	100.98	0.9726	99.47	0.9401
			R-ACTGGATACCCTTTCGCAGA						
*TUA*	Alpha tubulin	NM_121982	F-ACTTGGCTTGCTGTTTGATG	159	59.2/59.0	93.82	0.9831	96.14	0.9969
			R-CAGTTGGTGGCTGGTAGTTG						
*UBQ9*	Ubiquitin 9	NM_118934.3	F-CATCTTGAAGGAGCAGTGGA	152	59.0/59.0	98.84	0.9252	95.08	0.9855
			R-CAGTGGACTCGTACTTGTTCTTG						

*E- PCR efficiency

### Quantitative real time PCR (qRT -PCR)

qRT- PCR was performed using SYBR green technology in 96 well optical plate with lightcycler 480 real time PCR machine (Roche) adopting following thermocycle conditions: 95°C for 5min, 40 cycles of 95°C for 20 sec, 60°C for 60 sec and 72°C for 30 sec. Melt curve analysis was performed at 65–95°C at the end of the PCR run. PCR reaction mixture was prepared at a final volume of 20μl which contains 10 μl of TB Green Premix Ex Taq II) (Takara), 10pM of forward and reverse primers and 2μl of diluted cDNA. NTC (no template control) was maintained in every run for all the RGs. Three technical replicates were maintained for all the experiment. Ct values were recorded and utilized further for analysis.

### Data analysis

Descriptive statistical analysis was performed using Microsoft Excel 2016. The measurement of expression stability of seven RGs across 128 samples of each genotype was conducted with the help of three statistical software geNorm, Normfinder and BestKeeper. Ct value obtained by qRT-PCR was transformed to relative expression level for each RG with the help of delta-Ct method which was then used in geNorm and Normfinder for further analysis. Original Ct values obtained were used for the analysis in BestKeeper software.

## Results

### Amplification specificity and primer efficiency analysis of candidate reference genes

**Fig 1:**
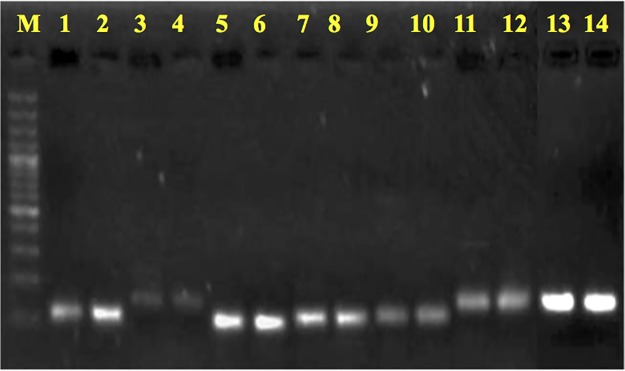
Confirmation of expected amplicon size for each primer pair. 2% agarose gel electrophoresis showing amplicon of a single product of expected size in both the genotypes for each reference gene. From left to right: M– 100 bp ladder, 1- *ACT7* (*B*. *Juncea*), 2-*ACT7* (*C*.*sativa*), 3- *CAC* (*B*. *juncea*), 4- *CAC* (*C*.*sativa*), 5- *EF1A* (*B*. *juncea*), 6- *EF1A*(*C*. *sativa*), 7- *PP2A* (*B*. *juncea*), 8- *PP2A* (*C*. *sativa*), 9- *TIPS41* (*B*. *juncea*), 10- *TIPS41* (*C*.*sativa*), 11-*TUA* (*B*. *juncea*), 12- *TUA* (*C*. *sativa*), 13- *UBQ9* (*B*. *juncea*), 14- *UBQ9* (*C*.*sativa*).

PCR amplification specificity of seven reference genes was evaluated in both the genotypes with 2% agarose gel electrophoresis and melting curve analysis following qRT-PCR. Results of gel run indicated that all the reference genes primer amplified products of expected size and no dimers or non-specific amplification was observed (Fig 1). Melting curve analysis corroborated the same results by yielding single peak for all the RGs in both the genotypes under investigation (S1 and S2 Figs). Tenfold serially diluted cDNA was used to generate a standard curve and slopes of the standard curves were used to calculate correlation coefficient R^2^ and PCR efficiency of each primer in two genotypes (S3 and S4 Figs). In *B*. *juncea* the linear R2 value and PCR efficiency range from 0.9726 to 0.9994 and 93.82% to 100.98% respectively and in *C*. *sativa* the R2 value and PCR efficiency range from 0.91 to 0.9969 and 96.14% to 103.83% respectively (Table 1).

### Expression profiling of reference genes in *B*. *juncea* and *C*. *sativa*

**Fig 2:**
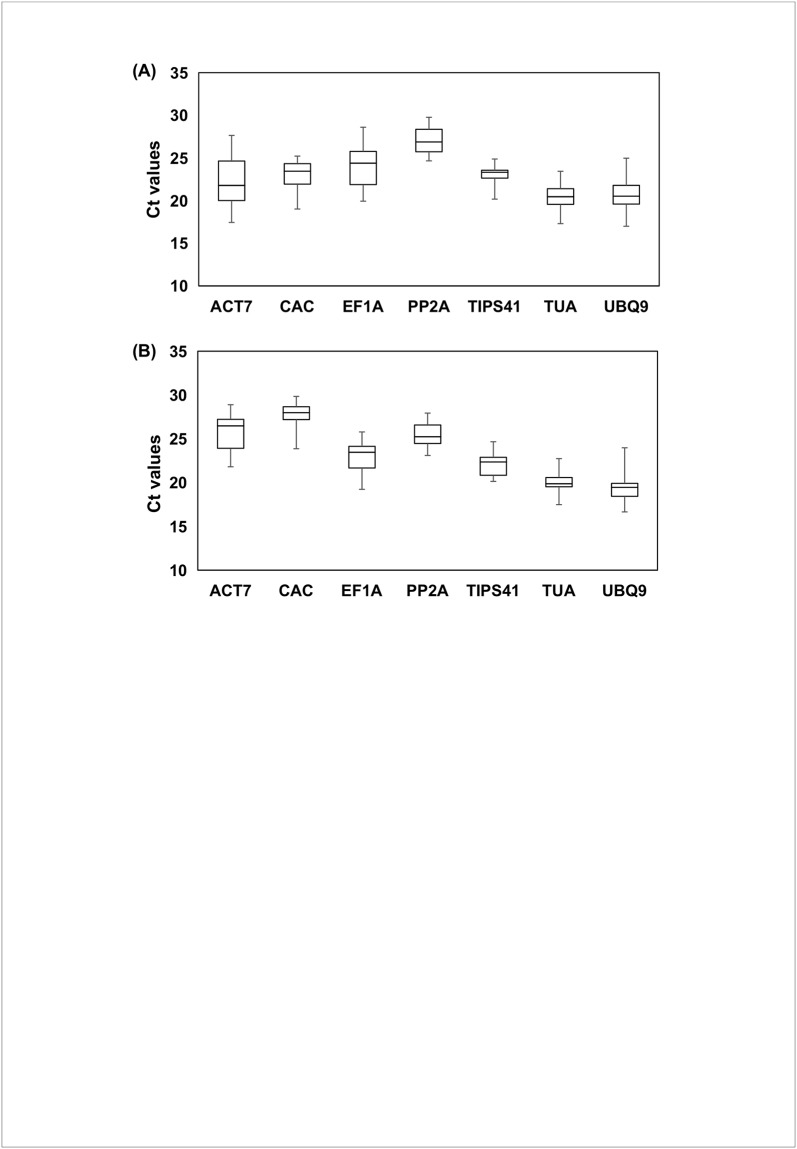
Expression levels of seven candidate RGs across all the experimental sets. (A) *B*. *juncea* (B) *C*. *sativa*. Solid line in the middle of the boxes represents median, and the boxes represents 25^th^ and 75^th^ percentile. Whiskers indicate the maximum and minimum values.

qRT-PCR assay based on SYBR green detection chemistry was designed for transcript profiling of seven RGs (*ACT7*, *CAC*, *EF1A*, *PP2A*, *TIPS41*, *TUA*, *UBQ9*) in two species. The expression levels of all the RGs were evaluated in 64 sample sets of each genotype subjected to eight different treatments (SA, MeJA, ABA, Wounding, *A*. *brassicae*, SA+ *A*. *brassicae*, JA+ *A*. *brassicae* and ABA+ *A*. *brassicae*) using threshold cycle (C_t_) values. In both the genotypes, a relatively wide range of C_t_ values was observed suggesting a diverse transcript abundance of all the RGs (Fig 2). In *B*. *juncea*, *TUA* was the most abundant reference gene (mean C_t_-20.43) of the set whereas, *PP2A* exhibited lowest abundant expression (mean Ct-27.02) across all the samples. Three novel reference genes *TIPS41*, *PP2A* and *CAC* showed lower gene expression variation (Coefficient of variation, CV of 4.54, 5.16 and 6.63% respectively) than the four traditionally adopted reference genes *TUA* (CV-6.75%), *UBQ9* (CV-9.58%), *EF1A* (CV-9.70%) and *ACT* (CV-12.56%) across all the samples. In the wild species *C*. *sativa*, *UBQ9* (mean C_t_-19.24) and, *CAC* (mean C_t_- 27.80) showed the most abundant and least abundant expression respectively across all the samples. As observed in *B*. *juncea*, in *C*. *sativa* also novel RG exhibited lower gene expression variation as compared to traditional genes. *CAC* (CV- 4.48%) showed least variation in expression across the samples. These results indicated a variable expression level of the RGs across all the samples subjected to different treatments in both the species. Therefore, it is necessary to evaluate and identify one or more RG(s) for accurate normalization under a defined set of experimental condition involving one or more of the genotypes adopted in the study.

### Gene expression stability and ranking of RG in *B*. *juncea* and *C*. *sativa*

**Fig 3:**
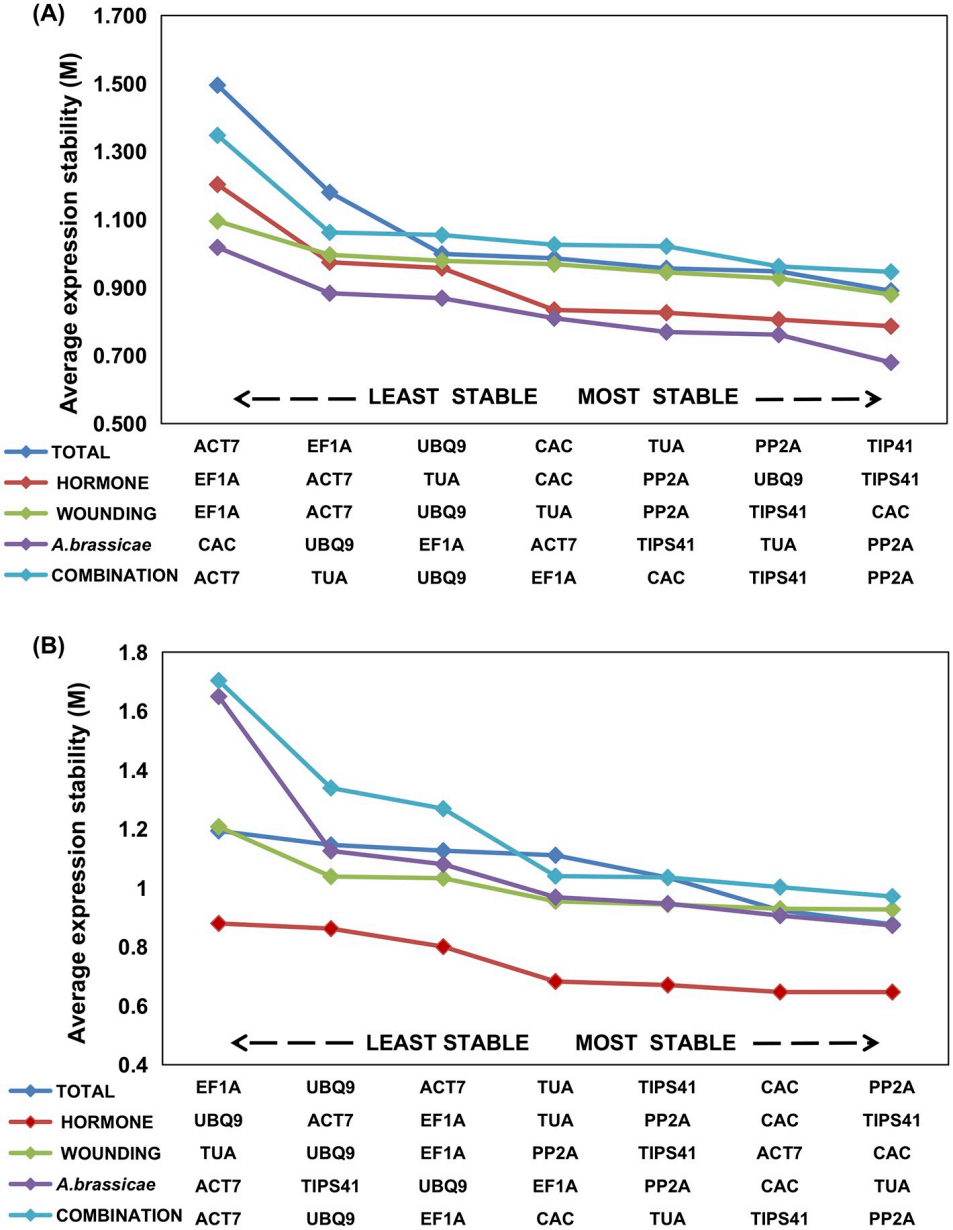
Average expression stability and ranking of seven reference gene predicted by geNorm. (A) *B*. *juncea* (B) *C*. *sativa*. Average expression stability value (M) is calculated by stepwise exclusion of the least stable reference gene across each experimental set. Lowest M value represents most stable reference gene, while highest value indicates unstable expression.

The expression stability of RGs in *B*. *juncea* and *C*. *sativa* was further assessed using three analytical programs namely geNorm, Normfinder and BestKeeper. 128 samples of each genotype were further divided into five groups: Hormone treatment (SA, MeJA and ABA), wounding, *A*. *brassicae* infection, combination treatment (hormone treatment followed by fungal inoculation) and total (all samples data included) and most stable RG were identified for each experimental set in both the genotypes. Based on geNorm analysis, across all the samples of *B*. *juncea TIPS41* (M value-0.890), *PP2A* (M value-0.948), and *TUA* (M value-0.956) were the three most stable reference genes. For hormone-treated and wounded samples, *TIPS41* and *CAC* exhibited most stable expression with an M value of 0.786 and 0.879 respectively. *PP2A* exhibiting M value of 0.679 for *A*. *brassicae* inoculated and 0.946 for combination-treated samples was the most stable reference gene in both the conditions (Fig 3a). In Wild genotype *C*. *sativa*, *PP2A* (M value 0.876), *CAC* (M value 0.923) and *TIPS41* (M value-1.035) showed maximum expression stability across all the samples. Whereas, *TIPS41*(M value 0.644), *CAC* (M value 0.926), *TUA* (M value-0.872) and *PP2A* (M value 0.971) showed most stable expression in hormone, wounding, *A*. *brassicae* and combination treatment group respectively (Fig 3b).

The tested data was also analyzed using Normfinder and BestKeeper software to further validate the geNorm analysis results. Ranking of reference genes generated by Normfinder and BestKeeper across all the samples of *B*. *juncea* and *C*. *sativa* was found closely similar to the ranking given by geNorm program with minor differences. The expression stability and ranking of RGs based on Normfinder and the BestKeeper software analysis across various sample sets in *B*. *juncea* is depicted in Table 2. In *B*. *juncea*, Normfinder identified *PP2A* and *TIPS41* as the two most stable reference gene in wounded and *A*. *brassicae* inoculated sample set. *UBQ9* and *TIPS41* were identified as the best reference gene for hormone and combination treatment samples respectively. Similarly, the expression stability and ranking of RGs across all the sample sets of *C*. *sativa* was also identified by Normfinder and BestKeeper and is depicted in Table 3. Normfinder identifies *CAC* as the most stable candidate RG in hormone and combination treatment samples whereas, in wounding and *A*. *brassicae* treatment *TIPS41* and *TUA* respectively were identified as the most stable transcript. BestKeeper mostly identified same top three candidate RG in most of the sample sets with a few exceptions like in the wounded samples of *C*. *sativa* NormFinder and geNorm identified *ACT7* as the second and third best RG respectively but BestKeeper ranked *ACT7* as the second worst gene in the same sample set. To identify the most suitable RGs in the respective experimental sets of each species and to identify the RGs which showed comparably stable expression in both the species the ranking given by all three programs were compared. The three best candidates identified from each program were considered and the common RG identified in all three programs were designated as the best suitable RGs for normalization of real time expression in both the species (Table 4).

**Table 2:** Expression stability and ranking of RGs in *B*. *juncea* under different experimental conditions identified by Normfinder and BestKeeper software.

Treatments	NormFinder			BestKeeper			
	RGs	Stability	Rank	RGs	Std Dev	Rank	r
Hormone treated	UBQ9	0.198	1	CAC	0.26	1	0.864
	TIPS41	0.219	2	UBQ9	0.37	2	0.800
	PP2A	0.259	3	TIPS41	0.42	3	0.910
	CAC	0.259	4	PP2A	0.52	4	0.732
	TUA	0.299	5	ACT	0.57	5	0.605
	ACT7	0.312	6	TUA	0.70	6	0.587
	EF1A	0.426	7	EF1A	0.86	7	0.671
Wounding	PP2A	0.289	1	PP2A	0.40	1	0.877
	TIPS41	0.304	2	UBQ9	0.45	2	0.972
	TUA	0.324	3	TIPS41	0.56	3	0.731
	UBQ9	0.366	4	CAC	0.68	4	0.899
	CAC	0.383	5	TUA	0.75	5	0.720
	ACT7	0.428	6	ACT	0.82	6	0.755
	EF1A	0.503	7	EF1A	0.85	7	0.772
*A*. *brassicae* inoculated	PP2A	0.126	1	TIPS41	0.50	1	0.941
	TIPS41	0.185	2	PP2A	0.53	2	0.871
	TUA	0.247	3	TUA	0.65	3	0.917
	UBQ9	0.260	4	UBQ9	0.69	4	0.812
	EF1A	0.321	5	CAC	0.70	5	0.755
	ACT7	0.336	6	EF1A	1.04	6	0.657
	CAC	0.515	7	ACT	1.20	7	0.877
Combination treatment	TIPS41	0.174	1	PP2A	0.47	1	0.863
	PP2A	0.276	2	TIPS41	0.56	2	0.888
	CAC	0.288	3	CAC	0.83	3	0.904
	EF1A	0.354	4	ACT7	1.08	4	0.732
	TUA	0.410	5	TUA	1.19	5	0.737
	UBQ9	0.442	6	EF1A	1.22	6	0.630
	ACT7	0.444	7	UBQ9	1.77	7	0.902
All samples	TIPS41	0.210	1	TIPS41	0.56	1	0.887
	PP2A	0.302	2	CAC	0.82	2	0.929
	TUA	0.307	3	PP2A	0.83	3	0.808
	CAC	0.324	4	EF1A	1.00	4	0.603
	UBQ9	0.327	5	UBQ9	1.07	5	0.694
	EF1A	0.368	6	TUA	1.19	6	0.642
	ACT7	0.427	7	ACT7	1.36	7	0.503

**Table 3:** Expression stability and ranking of RGs in *C*. *sativa* under different experimental conditions identified by Normfinder and BestKeeper software.

Treatments	NormFinder			BestKeeper			
	RGs	Stability	Rank	RGs	Std Dev	Rank	r
Hormone treated	CAC	0.160	1	CAC	0.23	1	0.912
	TIPS41	0.184	2	PP2A	0.31	2	0.885
	PP2A	0.230	3	TIPS41	0.48	3	0.749
	TUA	0.242	4	EF1A	0.58	4	0.632
	EF1A	0.249	5	UBQ9	0.62	5	0.683
	ACT7	0.270	6	TUA	0.71	6	0.812
	UBQ9	0.288	7	ACT7	0.78	7	0.602
Wounding	TIPS41	0.193	1	CAC	0.55	1	0.900
	CAC	0.197	2	PP2A	0.62	2	0.872
	ACT7	0.241	3	TIPS41	0.64	3	0.980
	UBQ9	0.253	4	UBQ9	0.77	4	0.784
	PP2A	0.275	5	TUA	0.79	5	0.889
	TUA	0.306	6	ACT7	1.32	6	0.879
	EF1A	0.337	7	EF1A	1.89	7	0.786
*A*. *brassicae* inoculated	TUA	0.185	1	CAC	0.36	1	0.910
	CAC	0.257	2	PP2A	0.39	2	0.879
	PP2A	0.258	3	TUA	0.54	3	0.845
	TIPS41	0.283	4	TIPS41	0.58	4	0.765
	UBQ9	0.335	5	UBQ9	0.75	5	0.793
	EF1A	0.343	6	ACT7	1.47	6	0.682
	ACT 7	0.530	7	EF1A	1.92	7	0.591
Combination treatment	CAC	0.253	1	TUA	0.20	1	0.863
	PP2A	0.275	2	TIPS41	0.40	2	0.888
	TUA	0.314	3	PP2A	0.47	3	0.904
	TIPS41	0.318	4	CAC	0.50	4	0.732
	EF1A	0.475	5	ACT7	0.55	5	0.637
	UBQ9	0.546	6	UBQ9	0.82	6	0.530
	ACT7	0.577	7	EF1A	1.32	7	0.902
All samples	PP2A	0.238	1	TUA	0.85	1	0.874
	CAC	0.304	2	PP2A	0.92	2	0.746
	TIPS41	0.370	3	TIPS41	0.94	3	0.827
	TUA	0.418	4	CAC	0.95	4	0.911
	ACT7	0.456	5	UBQ9	1.02	5	0.674
	UBQ9	0.482	6	EF1A	1.59	6	0.589
	EF1A	0.486	7	ACT7	1.61	7	0.678

**Table 4:** Reference genes identified based on results of geNorm, NormFinder and BestKeeper showing stable expression in *B*. *juncea* and *C*. *sativa*.

Sample sets	Three most stable RG of *B*. *juncea*	Three most stable RG of *C*. *sativa*	RG showing stable expression in both the species
Total	TIPS41, PP2A, TUA/CAC*	PP2A, TIPS41, CAC/TUA*	PP2A, TIPS41
Hormone	TIPS41, UBQ9, PP2A/CAC*	TIPS41, CAC, PP2A	TIPS41, PP2A
Wounding	TUA/ UBQ9*, PP2A, TIPS41	CAC, ACT7/PP2A*, TIPS41	TIPS41, PP2A
*A*. *brassicae*	PP2A, TUA, TIPS41/CAC**	TUA, CAC, PP2A	PP2A, TUA
Combination	PP2A, TIPS41, CAC/TUA*	PP2A, TUA, CAC/TIPS41^#^	PP2A, TUA

* RG identified among the three best RGs by BestKeeper but not by NormFinder and geNorm

**RG identified among the three best RGs by BestKeeper and Normfinder but not by geNorm.

^#^ RG identified among the three best RGs by BestKeeper and geNorm but not by Normfinder.

### Minimum number of candidate reference genes for accurate normalization in both the species

**Fig 4:**
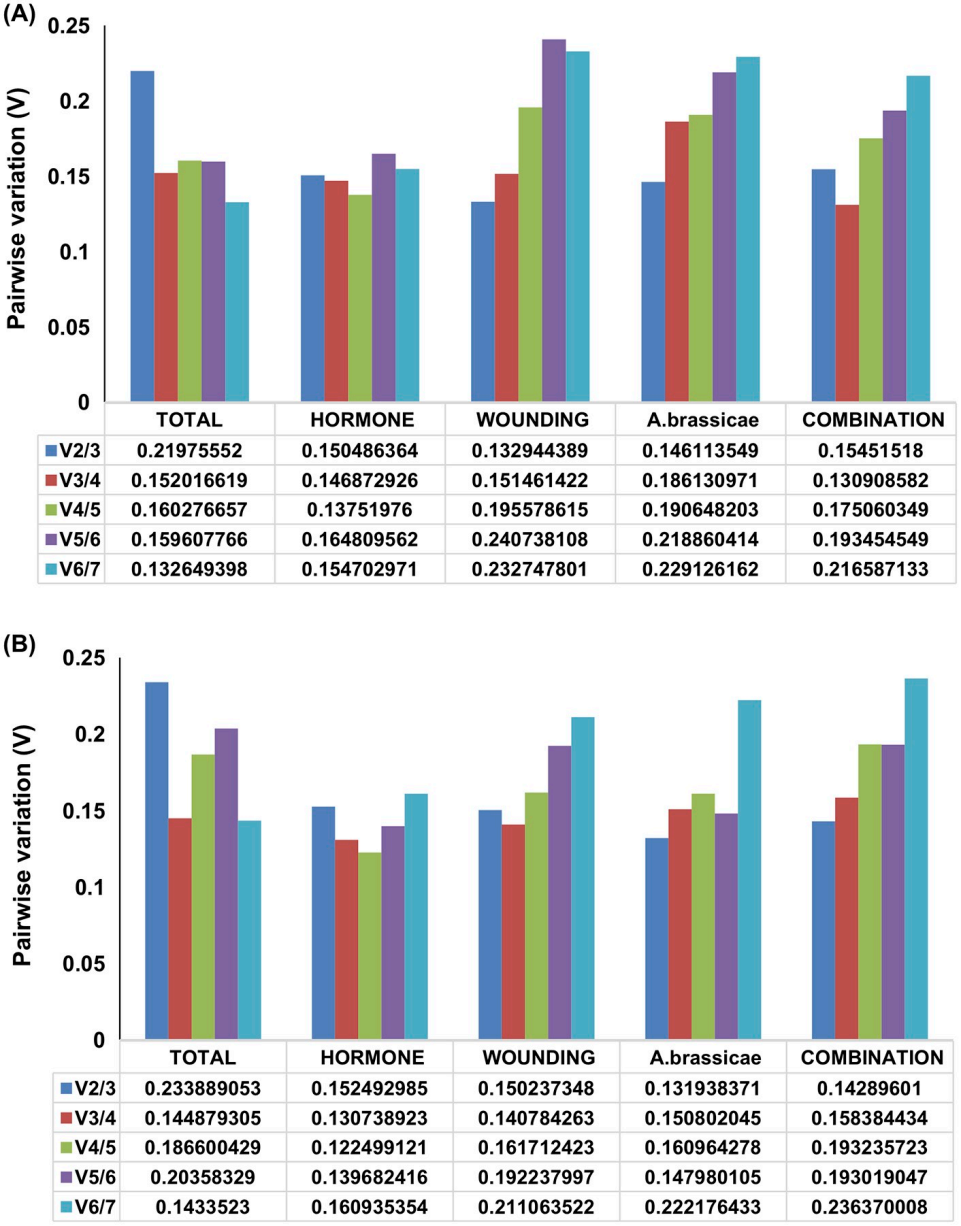
Determination of optimum number of reference genes for an accurate normalization. (A) *B*. *juncea* (B) *C*. *sativa*. Pairwise variation (V_n_/ V_n+1_) analysis of 7 reference gene in each experimental set.

The minimum number of RGs required for accurate normalization across different experimental sets in *B*. *juncea* and *C*. *sativa* was determined through geNorm program. The pairwise variation (V_n_/ V_n+1_) is calculated between the sequential normalization factor NF_n_ and NF_n+1_ in order to reveal the effect of adding (n+1) ^th^ gene in the analysis over the stability of normalization factor. Vandesomple *et al* 2002 recommended cutoff value of 0.15, beyond which adding new genes has no effect on expression stability value. As shown in (Fig 4), the difference in the expression stability value of RGs for hormone-treated sample was less marked than other sample sets in both the genotypes. Pairwise variation V2/3 value for the total sample set of *B*. *juncea* and *C*. *sativa* was 0.219 and 0.233 respectively. Applying the strict cutoff value (V = 0.15), at least three RGs must be included in the expression analysis when all treatment samples are considered together in both the genotypes. In the remaining experimental sets of both the genotypes, it was observed that V 2/3 value is less than 0.15 therefore, two most stable RGs are sufficient to perform an accurate normalization of qRT- PCR analysis. Based on the expression stability and the ranking scored of the RGs in both the genotypes we have identified suitable RGs that can be adopted in an expression analysis including either *B*. *juncea* or *C*. *sativa* or both the genotypes (Table 4). As depicted in Table 4, for the expression analysis involving individual genotype, two most stable RGs identified for each sample can be used as the ideal set RGs whereas, an expression study adopting C. *sativa* as a comparative yardstick against *B*. *juncea* the ideal set of RGs comprise of the two common candidate RGs out of three most stable reference genes in both the genotypes. For example, *TIPS41*, *UBQ9* and *PP2A* was identified as three most stable expressing RGs in hormone treated sample set of *B*. *juncea* and *CAC*, *UBQ9* and *TIPS41* as three best RGs in the same sample set of *C*. *sativa*. Therefore, we concluded that *PP2A* and *TIPS41* can be suitably used as RGs in comparative gene expression analysis of the target gene among these two crucifers.

### Reference gene validation in *B*. *juncea* and *C*. *sativa*

**Fig 5:**
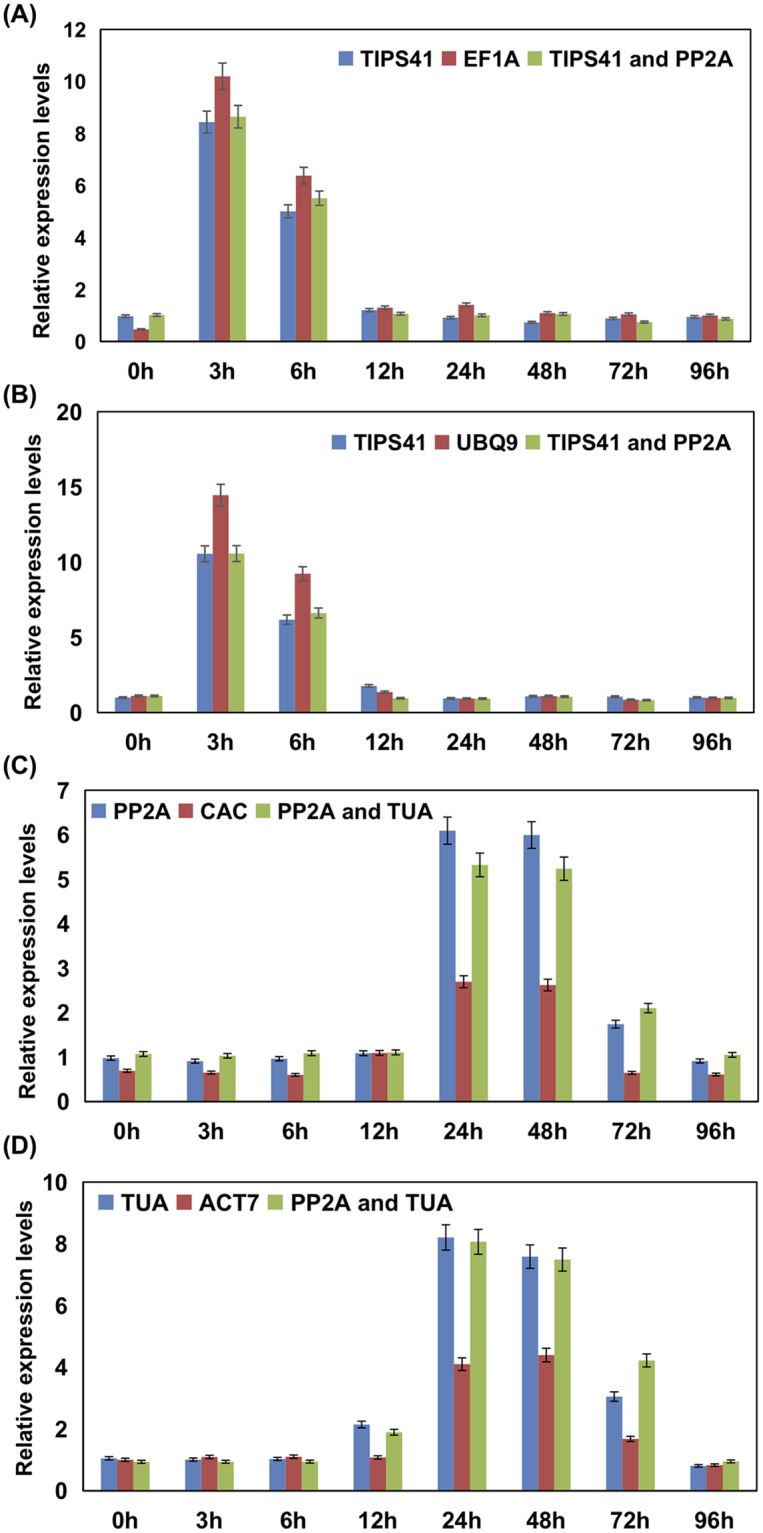
Validation of the identified reference genes. Relative quantification of *PDF1*.*2* in JA treated samples of (A) *B*. *juncea* (B) *C*. *sativa* and *A*. *brassicae* inoculated samples of (C) *B*. *juncea* (D) *C*. *sativa* using one most stable, one least stable and two most stable reference genes identified in respective treatment groups.

Many previous studies have shown that the use of unsuitable RGs could generate deceptive expression pattern of the gene of interest [12]. To validate the RGs confirmed above, relative expression of *PDF1*.*2* was measured in JA treated and *A*. *brassicae* inoculated samples of *B*. *juncea* and *C*. *sativa* at different time intervals after treatment. *PDF1*.*2* is a reported marker gene for JA induced signaling and JA signaling exerts a major influence on plant response to necrotrophic pathogen like *A*. *brassicae* [30, 31]. For qRT-PCR analysis one most stable, one least stable and two most stable RGs stably expressing in both genotypes in the respective treatment group was used (Table 4). As shown in Fig 5A and 5B, *PDF1*.*2* showed highest induction at 3 hours after treatment (hat) in JA treated samples of both the genotypes. When the expression level of *PDF1*.*2* was calculated in JA treated samples of *B*. *juncea* and *C*. *sativa* with most stable RG (*TIPS41*) and with two stable RGs similar fold change patterns with non-significant variation were observed. Whereas, when the expression level of *PDF1*.*2* was evaluated with the least stable RGs *EF1A* and *UBQ9* in *B*. *juncea* and *C*. *sativa* respectively, an overestimation of the expression levels was observed in both genotypes. When the least stable RG was included in expression analysis *C*. *sativa* exhibited approximately four-fold higher expression of *PDF1*.*2* as compared to *B*. *juncea* 3 hrs after JA treatment. On the other hand, when *TIPS41* and combination of two stable RGs (*TIPS41* and *PP2A*) were used the expression level of *PDF1*.*2* was found to be approximately two-fold higher in *C*. *sativa* than *B*. *juncea*. In *A*. *brassicae* inoculation, *PP2A* and *CAC* were identified as most stable and least stable RG respectively in *B*. *juncea*. Whereas, in *C*. *sativa TUA* and *ACT7* were identified as most stable and least stable RG respectively. *PP2A* and *TUA* exhibited comparable and stable expression in both the genotypes. As depicted in Fig 5C and 5D, *C*. *sativa* exhibits a higher induction of *PDF1*.*2* as compared to *B*. *juncea* in response to *A*. *brassicae*. Highest gene induction was achieved 24 hrs post inoculation (hai) in both the genotypes although, *C*. *sativa* showed a moderate increase in *PDF1*.*2* transcript level 12hpi and no such early induction was noticed in *B*. *juncea at the same time point*. In our study, using least stable RG for calculating relative expression of *PDF1*.*2* in *A*. *brassicae* sample set leads to an underestimation of expression levels in both the genotypes. Upon comparing the expression level of *PDF1*.*2* among *B*. *juncea* and *C*. *sativa*, using least stable RG for analysis no difference in the fold change was observed until *12 hai* and at *24 hai C*. *sativa* achieved a 1.4-fold higher gene expression as compared to *B*. *juncea*. Contrarily, using a stable RG and a combination of two most stable RGs detected approx. 1-fold higher gene expression in *C*. *sativa 12 hai* which remained undetected when least stable RG was used for normalization of gene expression analysis. Further, *B*. *juncea exhibited* 2.5-fold higher expression of *PDF1*.*2* as compared to *C*. *sativa* at *24 hai*.

## Discussion

qRT-PCR is a sensitive, high throughput and accurate technique for gene expression analysis which provide important insights to the plant biological processes [2,32]. The accuracy of gene expression analysis is largely influenced by the expression stability of the RG used for the normalization [33,34]. In recent years, many studies have been performed under various abiotic and biotic stress conditions for the selection of suitable RGs in *B*. *juncea* [14] and its cenospecies [7–9, 13]. However, until now no such systematic study for the validation of RGs has been conducted for *C*. *sativa*. A comparative gene expression analysis between two closely related species exhibiting variable response towards stress is a common approach to understand the molecular mechanism of stress responses in plants. Selecting an inappropriate RG can lead to misinterpretation of the expression level of the target genes. In this study, we have identified stable RGs for accurate comparative expression analysis in these two closely related species.

In this study we sought suitable RGs for expression analysis in *B*. *juncea* and *C*. *sativa* under diverse experimental conditions including hormone treatment, wounding, *A*. *brassicae* inoculation and combination treatment (hormone followed by *A*. *brassicae*). In a previous report on *B*. *juncea*, suitable RGs for hormone-treated and wounded samples were identified for an unbiased qRT-PCR analysis [14]. However, for *A*. *brassicae* treatment and combination treatment no such experiments have been conducted so far. geNorm, NormFinder and BestKeeper programs were used in our study and the results from these algorithms were largely similar, but they did not rank the genes in the exact same order. In our study the results from BestKeeper were slightly varied from the results of the other two software for example, BestKeeper identified *CAC* as the most stable RG in hormone treated samples of *B*. *juncea* but the other two software identified *CAC* as the fourth best gene for the same sample sets. This variation occurs because geNorm and Normfinder identifies most stable RGs by analyzing the pairwise variation between two RGs but Bestkeeper analyzes the stability of each gene individually. Some previous reports have described that results of the BestKeeper are less precise as compared to geNorm and Norfinder because of its individual gene analysis approach. Therefore, we have compared the results of all three programs while determining the suitable RGs for a particular experimental set.

In order to select the suitable RGs for the comparative gene expression analysis in *B*. *juncea* and *C*. *sativa the RGs expressing stably in both the species under a given experimental condition is identified*. For example, In wounded samples of *B*. *juncea*, geNorm identified *CAC* as most stable RG and *TIPS41* and *PP2A* were ranked second and third respectively but NormFinder ranked *CAC* as fifth and *PP2A* as most stable gene followed by *TIPS41* and BestKeeper ranked *PP2A* as first and *TIPS41* as third most stable RG. Therefore, according to these results we recommended *TIPS41* and *PP2A* as the two most stable reference gene for wounded samples of *B*. *juncea*. In wounded samples of *C*. *sativa*, both geNorm and Normfinder identified *CAC*, *TIPS41* and *ACT7* as three most stable RGs and BestKeeper identified CAC, PP2A and TIPS41 as three best genes. Hence, we concluded that *TIPS41* is the most suitable RG for relative expression analysis of wounding treatment in *B*. *juncea* and *C*. *sativa*. Similarly, Genes identified consistently as the stable expressing RGs various sample sets of *B*. *juncea* and *C*. *sativa* were identified by comparing the results of all the program used for the analysis. These RGs can be used in the comparative gene expression analysis of the target genes in *B*. *juncea* and *C*. *sativa* to achieve an unbiased gene expression quantification.

Interestingly, the commonly used housekeeping genes *ACT7*, *EF1A*, *TUA* and *UBQ9* were found to be among the least stable transcript in most of the sample sets with a few exceptions, like *UBQ9* and *ACT7* showed 2^nd^ most stable expression in hormone-treated samples of *B*. *juncea and* wounded samples of *C*. *sativa* respectively. *TUA* showed stable expression in *A*. *brassicae* inoculated samples of both the genotypes. Poor expression stability of *ACT7* was also reported in many previous studies in Rice [11], potato [18] and *Arabidopsis* [12] under diverse condition and treatments. In the current study, in hormone-treated sample set of *B*. *juncea*, *UBQ9* was found to exhibit stable expression these results corroborated a previous report in *B*. *juncea* where *UBQ9* was identified as the best gene for normalization in hormone-treated samples [14]. However, in *C*. *sativa* hormone-treated samples both geNorm and NormFinder program identified of *UBQ9* as the least stable reference gene in our study and BestKeeper also ranked *UBQ9* among the least stable RGs in the respective sample set. Therefore, we do not recommend *UBQ9* as a suitable reference for comparative gene expression analysis of hormone-treated samples among *B*. *juncea* and *C*. *sativa*. Hence, it can be understood that there is no “universal reference gene” which can be used in all similar studies. Normalization analysis and validation of RG is important even when a gene has been proved to be stably expressed in the many related species and diverse experimental conditions.

In the current study, we explored the expression stability of novel reference gene *TIPS41*(Tonoplast intrinsic proteins), *PP2A* (protein phosphatase 2A) and *CAC* (Clathrin adaptor complex) in *B*. *juncea* and *C*. *sativa*. In our study, *TIPS41 and PP2A* consistently showed stable expression across all the experimental sets of *B*. *juncea*. A previous study identified *TIPS41* and *CAC* as the most stable RG in different treatments and tissue types of *B*. *juncea* [14]. In our experiment, *TIPS41* and *PP2A* exhibited higher expression stability as compared to *CAC* in *B*. *juncea*. However, in the study mentioned above [14] expression stability of *PP2A* was not analyzed. Therefore, based on our results we concluded that *PP2A* also performed at par with *TIPS41* in terms of expression stability in *B*. *juncea* under the given treatment conditions. In a related species *B*. *napus*, *PP2A* and *TIPS41* were identified as stable reference genes in diverse experimental conditions and tissues type [7,35]. In the wild genotype used in this study, *C*. *sativa*, *TIPS41* was among the best performing reference gene in all the experimental sets except for *A*. *brassicae* treated set where *PP2A* and *CAC* exhibited a stable expression. Altogether we concluded that *TIPS41* and *PP2A* showed the most stable expression across all the experimental sets in *B*. *juncea* and *C*. *sativa* and a combination of these reference genes can be used for accurate normalization of gene expression in *B*. *juncea and C*. *sativa* under the described experimental conditions. Our finding was supported by previous studies in *B*. *juncea* [14], *B*. *napus* [7] and *Arabidopsis* [13], Probably reflecting on the ability of *TIPS41* and *PP2A* to maintain a stable expression level across the *Brassica* crops irrespective of the treatments. For the validation of identified reference genes, we chose *PDF1*.*2* gene as a target gene and its relative expression was calculated in JA treated and *A*. *brassicae* infected samples of *B*. *juncea* and *C*. *sativa*. *PDF1*.*2*, an important JA marker gene which is induced by exogenous application of MeJA and shows a systemic accumulation post inoculation with necrotrophic pathogen [30,36,37]. The expression pattern analysis of *PDF1*.*2* in JA treated and *A*. *brassicae* inoculated samples emphasized on the importance of choosing the appropriate RG. When different RGs were adopted for gene expression analysis of *PDF1*.*2* it is clearly evident that using an inappropriate RG causes biased estimation of the gene expression levels of the target gene. For comparative gene expression study involving *B*. *juncea* and *C*. *sativa*, normalization using more than one reference gene which shows comparable expression stability in both genotypes is recommended.

## Conclusion

In the present study, we evaluated the expression stability of 7 candidate RGs across diverse sample sets of *B*. *juncea* and *C*. *sativa* in order to identify suitable RGs for normalization of gene expression analysis in both the genotypes. This study is the first systematic attempt to identify stable RGs in *C*. *sativa* under different treatments. For the comparative gene expression analysis involving *B*. *juncea* and its wild relative *C*. *sativa* our study recognized RGs showing stable expression in both genotypes in diverse experimental conditions. Analysis based on three program recognized *TIPS41* and *PP2A* as the two most stable reference across 64 sample sets of *B*. *juncea* and *C*. *sativa* subjected to different treatments. Expression pattern analysis of *PDF1*.*2* in JA treated and *A*. *brassicae* inoculated samples emphasized on the importance of selecting suitable reference gene for accurate qRT-PCR analysis. Our results suggested that novel RGs performed better than traditional housekeeping genes adopted commonly in qRT-PCR analysis. We conclude that the results summarized in our investigation will facilitate accurate quantification of target gene expression in *B*. *juncea* and *C*. *sativa*. Further, this study can facilitate accurate comparative gene expression analysis in the cultivated species *B*. *juncea* and its wild relative *C*. *sativa* for a better understanding of molecular mechanism associated with various abiotic and biotic stresses.

## Supporting information

S1 Fig Melt curve generated for seven candidate reference gene in *B*. *juncea* samples. (PPTX)

S2 Fig Melt curve generated for all the seven candidate reference gene in *C*. *sativa samples*. (PPTX)

S3 Fig Amplification efficiencies of the designed primers of all candidate reference gene in *B*. *juncea*. (PPTX)

S4 Fig Amplification efficiencies of the designed primers of all candidate reference gene in *C*. *sativa*. (PPTX)

S1 Doc List of accession number of *Arabidopsis*, *B*. *juncea* and *C*. *sativa* gene sequences used for the multiple alignment and primer designing. (DOCX)
