# Supplementary material for: Evaluation of suitable reference genes in Brassica juncea and its wild relative Camelina sativa for qRT-PCR analysis under various stress conditions
Source: PLoS One. 2019 Sep 20;14(9):e0222530. doi: 10.1371/journal.pone.0222530 (PMC6754150; doi:10.1371/journal.pone.0222530)
Supplement: S1 Fig — (PPTX) [file pone.0222530.s001.pptx]

## Slide 1
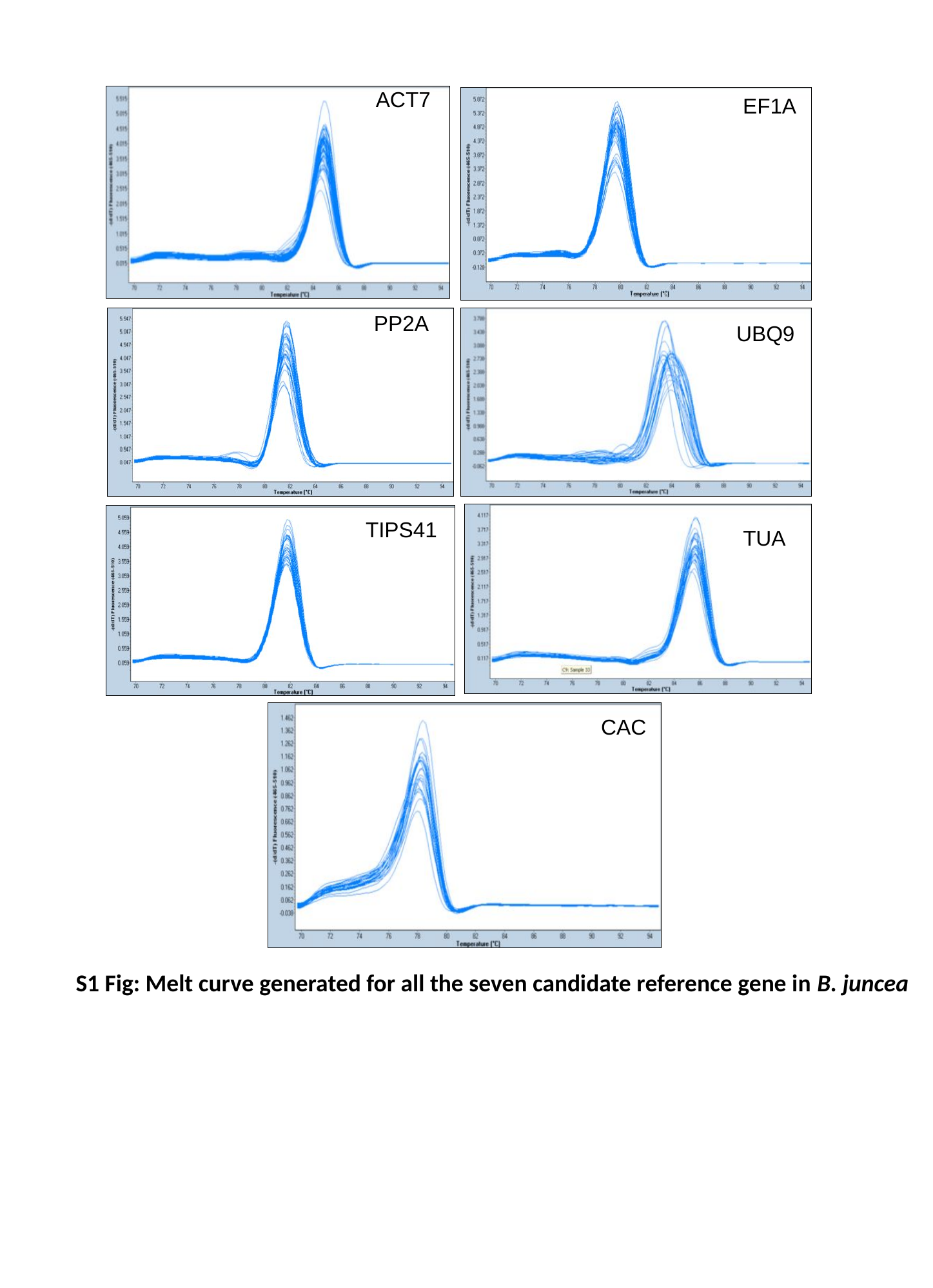

ACT7
EF1A
PP2A
UBQ9
TIPS41
TUA
CAC
S1 Fig: Melt curve generated for all the seven candidate reference gene in B. juncea
