# Supplementary material for: Evaluation of suitable reference genes in Brassica juncea and its wild relative Camelina sativa for qRT-PCR analysis under various stress conditions
Source: PLoS One. 2019 Sep 20;14(9):e0222530. doi: 10.1371/journal.pone.0222530 (PMC6754150; doi:10.1371/journal.pone.0222530)

## Slide 1
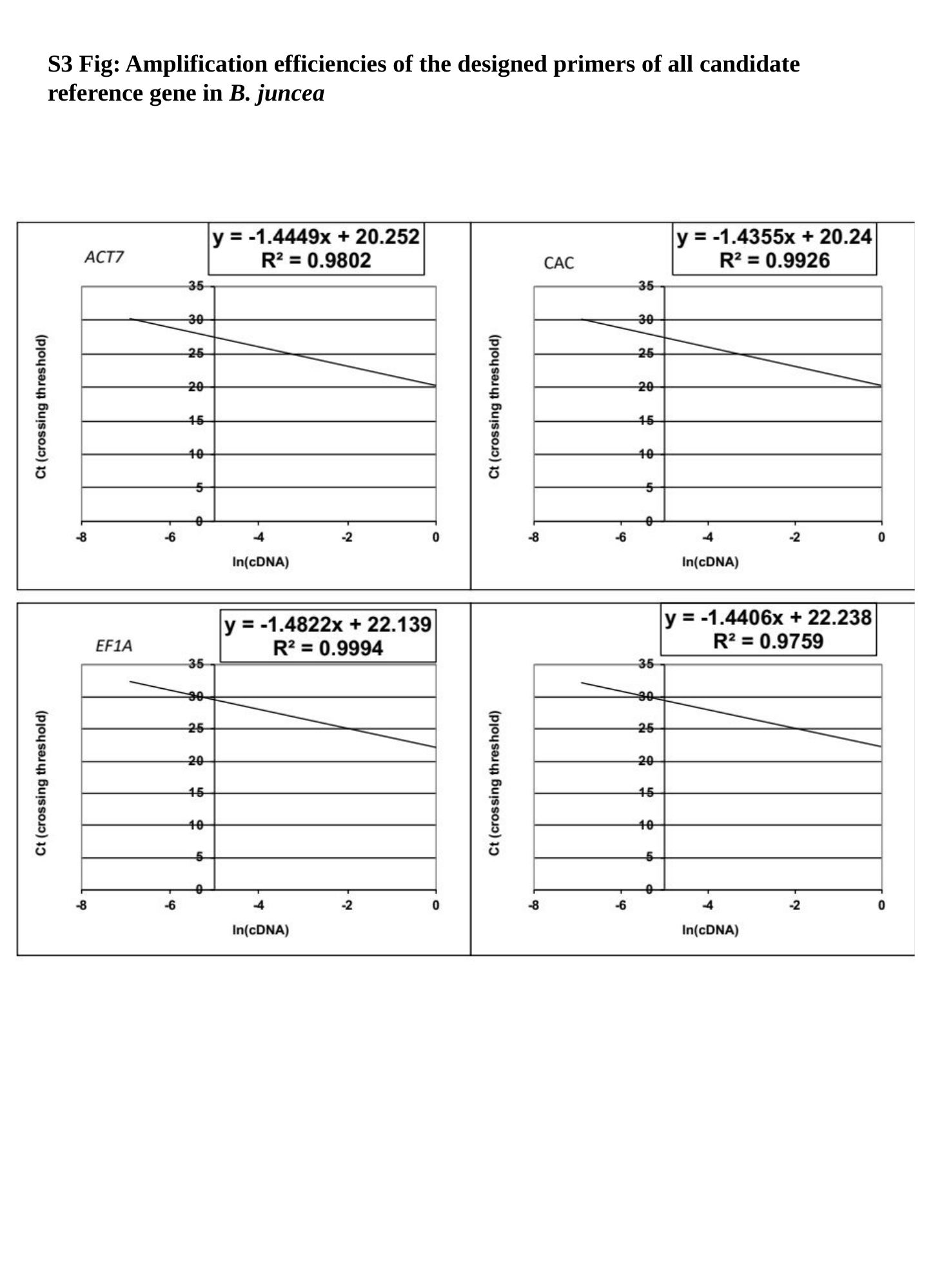

S3 Fig: Amplification efficiencies of the designed primers of all candidate reference gene in B. juncea

## Slide 2
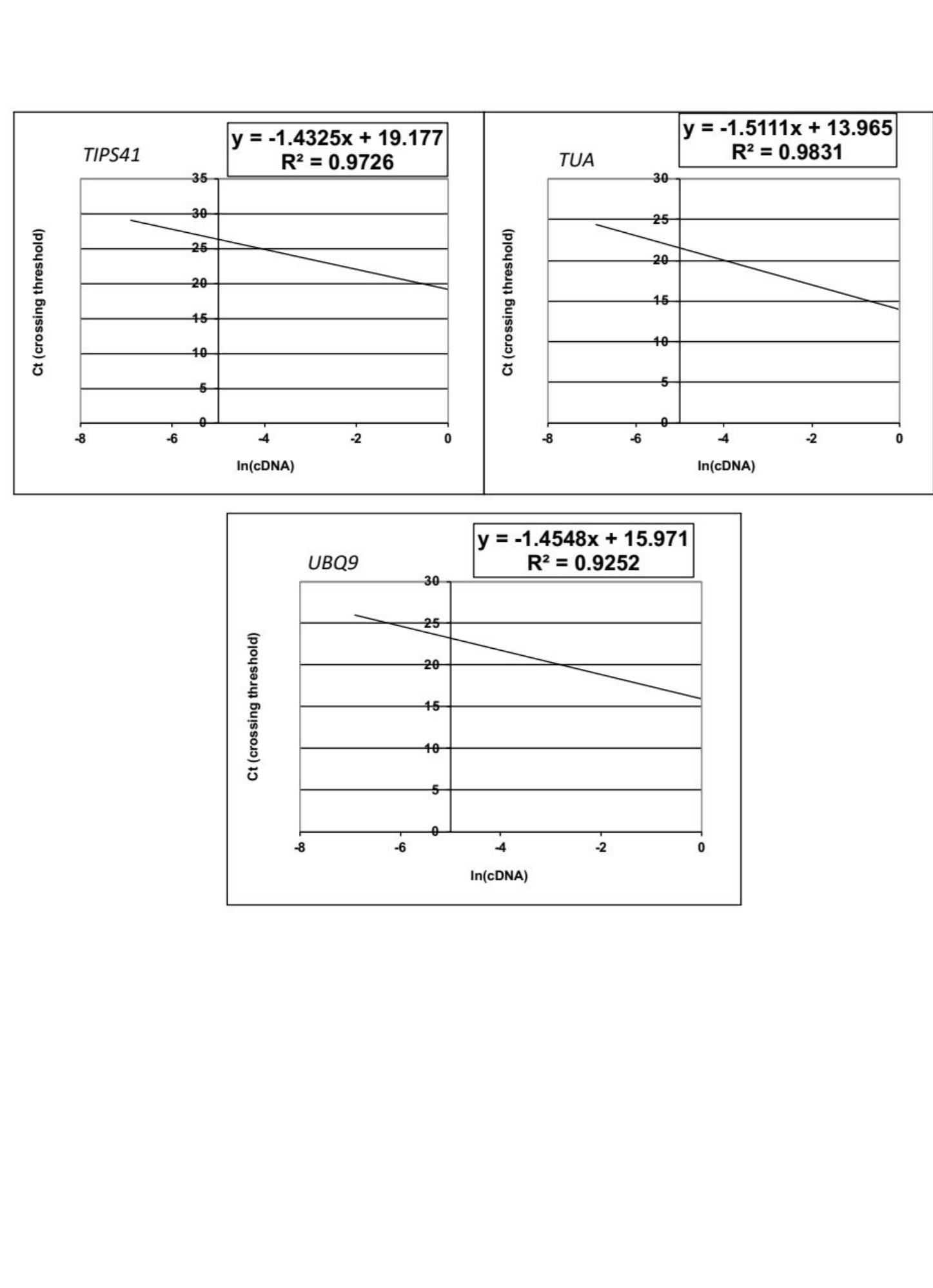

Supplement: S3 Fig — (PPTX) [file pone.0222530.s003.pptx]
