# Supplementary material for: Evaluation of suitable reference genes in Brassica juncea and its wild relative Camelina sativa for qRT-PCR analysis under various stress conditions
Source: PLoS One. 2019 Sep 20;14(9):e0222530. doi: 10.1371/journal.pone.0222530 (PMC6754150; doi:10.1371/journal.pone.0222530)

## Slide 1
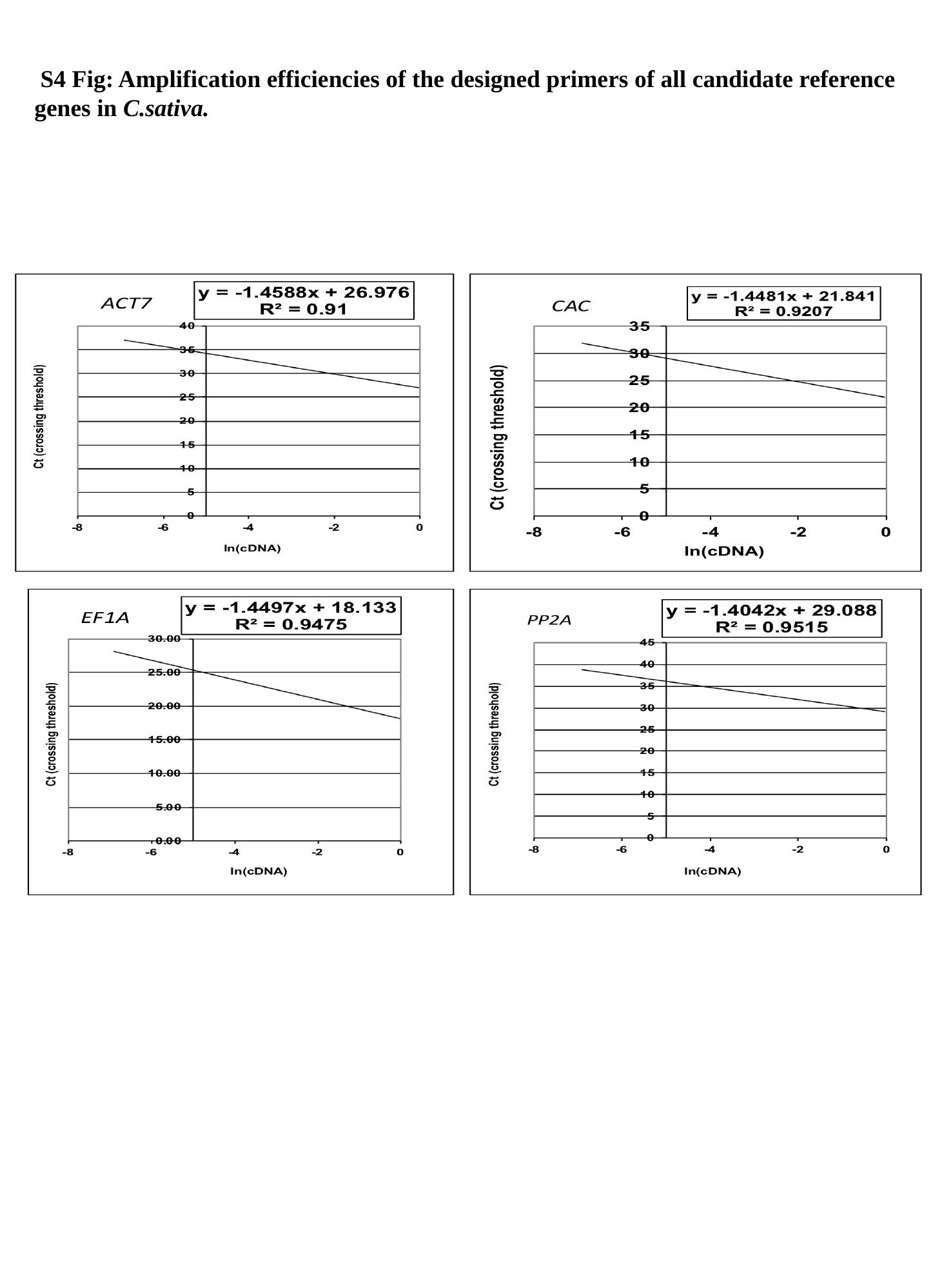

S4 Fig: Amplification efficiencies of the designed primers of all candidate reference genes in C.sativa.

## Slide 2
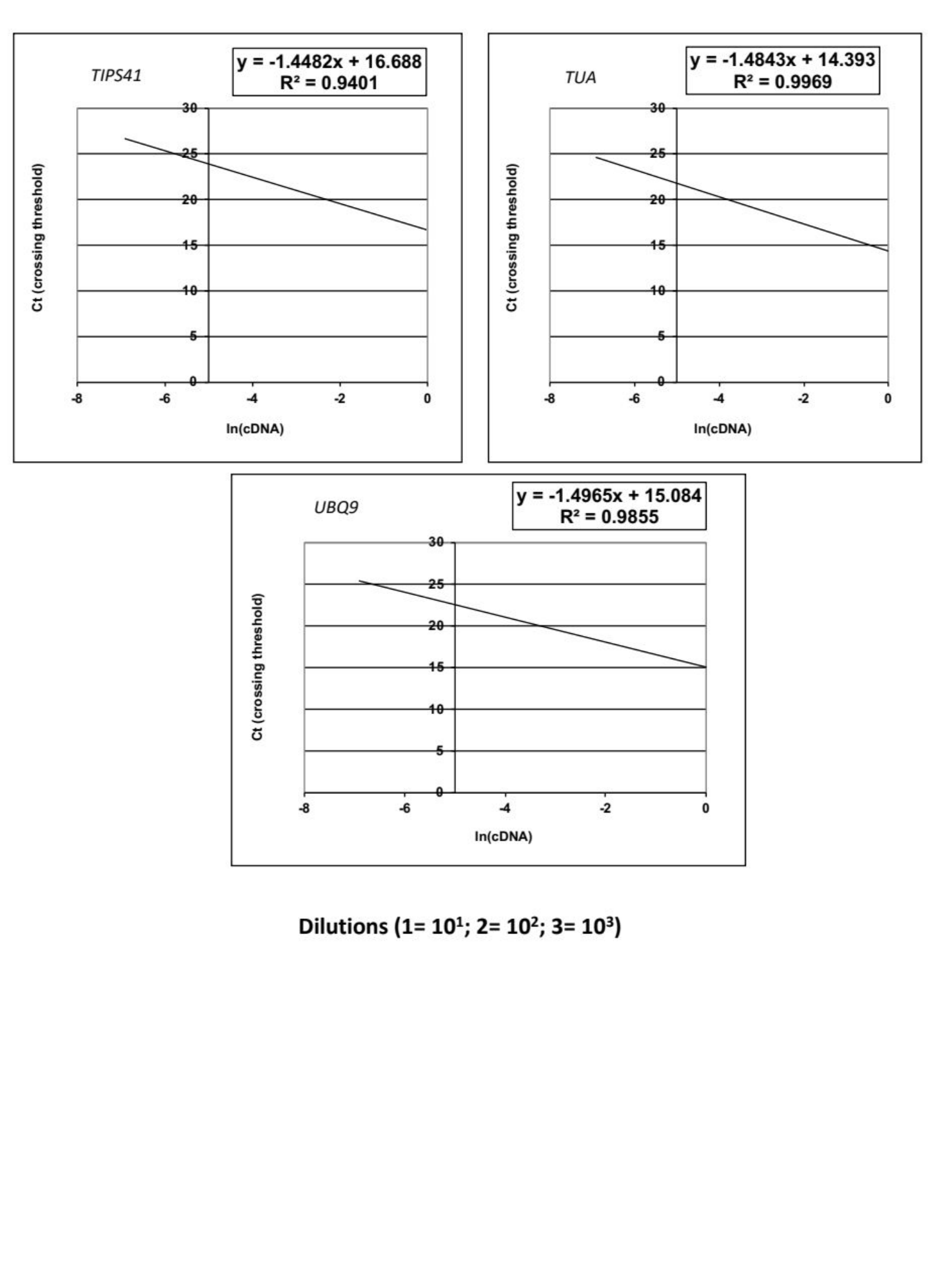

Supplement: S4 Fig — (PPTX) [file pone.0222530.s004.pptx]
