# Supplementary material for: Evaluation of suitable reference genes in Brassica juncea and its wild relative Camelina sativa for qRT-PCR analysis under various stress conditions
Source: PLoS One. 2019 Sep 20;14(9):e0222530. doi: 10.1371/journal.pone.0222530 (PMC6754150; doi:10.1371/journal.pone.0222530)
Supplement: S1 Doc — (DOCX) [file pone.0222530.s005.docx]

**Supplementary file S1. docx:** List of accession number of each genes of *Arabidopsis*, *B. juncea* and *C. sativa* used for the multiple alignment and primers were designed from the conserved region

| Gene | *Arabidopsis* accession | *B. juncea* accession | *C. sativa* accession |
| --- | --- | --- | --- |
| *ACT7* | NM_121018 | BjuB012485 | Csa08g054840 |
|  |  |  |  |
| *CAC* | NM_124033.4 | BjuB026945 | XM_010496541.2 |
|  |  |  |  |
| *EF1A* | XM_021015179 | BjuA006545 | Csa17g011360 |
|  |  |  |  |
| *PP2A* | BT000108 | BjuA030041 | Csa17g034980 |
| *TIPS41* | NM_119592.5 | Bra011516 | Csa12g009400 |
|  |  |  |  |
| *TUA* | NM_121982 | BjuA019283 | XM_010481098.1 |
|  |  |  |  |
| *UBQ9* | NM_118934.3 | BjuA006948 | Csa18g024310 |
